# Exploring predictors at toddler age of distinct profiles of attentional functioning in 6-year-old children born moderate-to-late preterm and full term

**DOI:** 10.1371/journal.pone.0254797

**Published:** 2021-07-29

**Authors:** Lilly Bogičević, Marjolein Verhoeven, Anneloes L. van Baar

**Affiliations:** Child and Adolescent Studies, Utrecht University, Utrecht, Netherlands; Clinic Hospital of Zaragoza, SPAIN

## Abstract

**Objective:**

Examining relationships of toddler abilities in attention, cognitive, motor, and language development, and behavioral problems, with distinct attention profiles at 6 years of age in children born moderate-to-late preterm and full term.

**Method:**

Longitudinal study with a cohort of 88 moderate-to-late preterm and 83 full term born children. At 18 months attention abilities were assessed. At 24 months cognitive, motor, and language development was examined and behavioral problems were screened. At 6 years ten measures of attention were administered, which were used to classify children in one of four attentional functioning profiles (normal attention, overall poorer attention, poorer cognitive attention, and behavioral attention problems). Performance at 18 and 24 months was examined in relation to these four distinct attention profiles, as well as in relation to normal (first profile) versus subaverage attention (second, third, and fourth profiles) using multinomial logistic regressions.

**Results:**

Orienting and alerting attention, and receptive language were related to distinct attention profiles. Specifically, children with an overall poorer attention profile at 6 years were differentiated by lower orienting attention and receptive language scores at toddler age, while those with a poorer cognitive attention profile showed lower early alerting attention at 18 months. Children with a behavioral attention problems profile at 6 years were differentiated by lower orienting attention but higher alerting attention scores at toddler age. Orienting attention and receptive language, but not alerting attention, at toddler age were related to normal versus subaverage attention, with lower scores predicting subaverage attention.

**Conclusions:**

Children at risk of poorer attentional functioning at school-age, expressed in distinct attention profiles, already showed differentiated functioning in attention abilities and in language comprehension as toddlers. Distinguishing distinct attention profiles could be important for future research and clinical practice, as is early monitoring of attention and language abilities in children at risk.

## Introduction

Attention represents a range of complex abilities. Cognitive abilities include orienting, shifting, achieving and sustaining, and planning and directing focus [1–4]. These abilities can be reflected in attention-related behaviors, such as (low) concentration and (in)attentiveness. Attention difficulties in childhood predict poorer academic and social functioning at later ages [5–8], and thus, early detection of children at risk of attention difficulties is essential for timely implementation of interventions.

Preterm birth is a well-known risk factor for poorer attentional functioning [9–15]. However, identification and treatment of children born preterm with attention difficulties is complicated, as they show heterogenous attentional outcomes [16–18]. In a previous study, we demonstrated variability in attentional functioning at 6 years in children born moderately-to-late preterm (MLPT; 32–36 weeks’ gestation) and full term (FT; 37–41 weeks’ gestation) [16]. We identified four distinct–one normal and three subaverage–profiles of attentional functioning across three attention domains (orienting attention & processing speed, alerting attention, and behavioral attention problems). All three subaverage attention profiles were characterized by poorer functioning in one or more attention domains, but differed in patterns of attentional functioning. Children born MLPT were twice as likely to have a subaverage attention profile and were more dispersed across the three subaverage attention profiles compared with FT children, of whom 80% had a normal attention profile [16]. As different types of attention difficulties are related to other skills–e.g. alerting attention and processing speed difficulties have been associated with poorer arithmetic, while only processing speed difficulties have been associated with poorer reading comprehension [19]–distinct attention profiles may be uniquely important for children’s functioning.

As especially children born MLPT exhibit various types of subaverage attention profiles, MLPT birth is an important, yet unspecific, marker for poorer attentional functioning. Therefore, other factors than preterm birth need to be considered to gain a better understanding of variation in attentional functioning. Early childhood abilities are building blocks for later cognitive functioning [20, 21]. Relatively general measures of early milestones in cognitive, motor, and language development, and measures of behavioral problems are commonly used to monitor development and may capture early abilities as important predictors for later functioning [15, 16, 22–24].

Specific measures of early attention abilities are also promising for predicting later cognitive outcomes and for differentiation of children at risk [9, 20, 21, 25–28]. Individual differences in attention appear continuous throughout development [29, 30]. Yet, previous studies have not examined toddler attention abilities in relation to school-age attentional functioning, and literature addressing early markers of variability in attentional functioning in particular is lacking. Studying toddler abilities in relation to distinct attention profiles may provide insight into the etiology of attention difficulties, enable earlier detection of children at risk of specific difficulties and identify potentially modifiable factors. Examining distinct attention profiles, as well as normal attentional functioning versus subaverage attentional functioning (the three subaverage profiles combined), could help identify early predictors for specific versus generalized attention difficulties.

Therefore, the aim of the current study was to explore if measures of attention, cognitive, motor, and language development, and behavioral problems at toddler age are predictive of a) four previously identified and distinct attention profiles [16], and b) normal attentional functioning versus subaverage attentional functioning at 6 years of age in a sample of children born MLPT and FT.

## Methods

### Participants and procedure

Participants were children from the longitudinal, prospective Study on Attention of Preterm children (STAP) Project. This study aimed to follow neurodevelopment, particularly attention, of children born MLPT in comparison with children born FT. Children were born between March 2010 and April 2011. At 10 months of age children were recruited from nine hospitals around Utrecht, the Netherlands. Children were excluded in case of dysmaturity, multiple births, admission to a tertiary Neonatal Intensive Care Unit, severe congenital malformations, antenatal substance abuse and chronic antenatal use of psychiatric drugs by the mother.

Children were evaluated at 18 months, 24 months, and 6 years of age by trained assessors who were blinded to birth status. MLPT children were invited at age corrected for prematurity, and normed cognitive scores were corrected for prematurity to minimize maturational effects and known bias in cognitive test scores [31]. Measures of attention were administered at 18 months, measures of cognitive, motor, language development, and measures of behavioral problems were administered at 24 months. Attentional functioning was assessed again at 6 years. The study was approved by the Utrecht Medical Center Ethics Committee and written informed consent was obtained from both parents.

### Measures

Table 1 presents the constructs, measurement tools and components for all measures.

**Table 1:** Overview of measures.

Variable type	Construct	Measurement tools	Components
Predictors	Attention at 18 months		
	Orienting attention	UTATE (E), orienting attention factor	Mean dwell time, transition rate, proportion of correct refixations, latency.
	Alerting attention	UTATE (E), alerting attention factor	Total dwell time, latency difference.
	Executive attention	UTATE (E), executive attention factor	Correct searches, mean delay.
	Joint attention	CIB (O), joint attention mean score	Mean score of children’s joint attention rated during 3 mother-child interaction tasks: a)
			free play, b) book reading, c) puzzle.
	Cognition, motor, language at 24 months	Bayley-III-NL (TB)	
	Cognition	Cognition scale	
	Fine motor	Fine motor scale	
	Gross motor	Gross motor scale	
	Receptive language	Receptive language scale	
	Expressive language	Expressive language scale	
	Behavioral problems at 24 months	CBCL/1.5–5 (M)	
	Emotionally reactive behavior	Emotionally reactive syndrome scale	
	Anxious or depressed behavior	Anxious/depressed syndrome scale	
	Somatic problems	Somatic problems syndrome scale	
	Withdrawn	Withdrawn syndrome scale	
	Sleep problems	Sleep problems syndrome scale	
	Attention problems	Attention problems syndrome scale	
	Aggressive behavior	Aggressive behavior syndrome scale	
Outcome measures	Attention profiles at 6 years	Latent profile analysis	Latent profiles derived from functioning across 3 attention domains: a) orienting attention
			& processing speed, b) alerting attention, c) behavioral attention problems.
	Normal vs. subaverage attention at 6 years	Dichotomization of attention profiles	Normal attention profile vs. 3 subaverage attention profiles combined.
Latent profile components	Orienting attention & processing speed	WPPSI (TB), COTAPP (TB),	Factor derived from EFA based on 6 attention measures.
		NEPSY (TB), TEA-Ch (TB)	
	Alerting attention	COTAPP (TB), TEA-Ch (TB)	Factor derived from EFA bases on 2 attention measures.
	Behavioral attention problems	CBCL/6-18 (M), TRF/6-18 (T)	Factor derived from EFA based on 2 attention measures.
Other variables	Neonatal characteristics		
	Medical characteristics		
	Sociodemographic characteristics		

TB = task-based, E = eye tracking, O = observational; M = mother-report, T = teacher-report.

WPPSI = Wechsler Preschool and Primary Scale of Intelligence, COTAPP = Cognitive Task Application, TEA-Ch = Test of Everyday Attention in Children, TRF/6-18 = Teacher Report Form 6–18.

EFA = exploratory factor analysis.

#### Attention at 18 months

The Utrecht Tasks of Attention in Toddlers using Eye tracking (UTATE) [32–34] was administered to evaluate orienting attention (i.e. engaging, disengaging, and shifting focus), alerting attention (i.e. maintaining focus for a considerable period), and executive attention (i.e. planning, directing, and inhibiting focus orienting) [3, 4]. Children were required to complete four tasks assessing various looking behaviors administered on an eye tracker with a total duration of 18 minutes: 1) the disengagement task, 2) the face task, 3) the alerting task, and 4) the delayed-response task. In the disengagement task first a visual stimulus was shown at the center of the screen, and after 2 seconds a second stimulus appeared at the left or right side of the central stimulus. This task consisted of 20 trials. In the face task first two identical photos of children’s faces were presented (habituation phase), and after 8.5 seconds, one of the photos changed into a new face. This new combination was then shown for another 8 seconds. This task consisted of eight trials. The alerting task consisted of 32 trials, in which a visual stimulus was presented. In half of the trials the stimulus was preceded by a sound (signal trials), while in the other the visual stimulus was not preceded by a sound (no-signal trials). In the delayed response task, the screen showed a dog that would hide in one of two doghouses. Once the dog was hidden, a worm appeared in the center of the screen to distract the child from the doghouses. After a delay the child was asked to search for the dog. This task consisted of 18 trials, in which the delay increased from 0 to 10 seconds in steps of 2 seconds after three consecutive trials. The tasks, including stimulus size and timing, are presented and described in more detail elsewhere [33]. The tasks yielded 13 variables which could be reduced to three latent constructs: orienting attention, alerting attention, and executive attention. Table 2 shows a description for each of these variables.

**Table 2:** Definitions of the variables from the UTATE.

Measure	Task	Definition
Orienting attention		
Mean dwell time	DIS, FACE	Average length of dwells. A dwell is the length of one visit in an area of interest (AOI).
Transition rate	DIS, FACE	The number of transitions (i.e. movement from one AOI to another) divided by the total dwell time.
Proportion of correct	DIS	A correct refixation indicates that the child refixated from the central stimulus to the new stimulus after the new
refixations		stimulus appeared. The proportion of correct refixations is the number of correct refixations divided by the
		total number of trials in which the child looked at the central stimulus when the new stimulus appeared.
Latency	DIS	The average time between appearance of the new stimulus and fixation on the new stimulus in trials in which the
Alerting attention		child correctly fixated.
Total dwell time	DIS, FACE, AL, DR	Sum of the length of all dwells.
Latency difference	AL	Difference between latencies in trials in which the stimulus appeared without signal (no-signal trials) and trials in
		which a signal preceded the appearance of the stimulus (signal trials).
Executive attention		
Correct searches	DR	The number of trials in which the child looked at the correct dog house directly responding to the voice asking the
		child to find the dog.
Mean delay	DR	The mean delay between hiding and the instruction to find the dog in which the child correctly searched for the dog.

DIS = disengagement task, FACE = face task, AL = alerting task, DR = delayed response task.

Based on the original confirmatory factor analysis [33], data from the total sample in the present study was used to repeat a confirmatory factor analysis with the same factor structure, with the R Project for Statistical Computing [35] and Lavaan package [36]. Model fit, assessed with RMSEA, CFI and TLI indices [37] was acceptable: *χ^2^* = 79.07, *P* = .002., RMSEA = .07, SRMR = .09, CFI = .96, TLI = .93. Scores on the three latent constructs of orienting, alerting, and executive attention were used, with higher scores indicating better attention capacities. The UTATE shows adequate to good reliability, and evidence for validity has been provided as well [33, 34].

The UTATE was performed using a Tobii T60 Eye Tracker with an integrated 17-inch TFT screen with a resolution of 1280 by 1024 pixels. The stimuli were presented on the screen using E-prime 2.0 software.

Mother-child interaction at 18 months was videotaped during a 10-minute structured play setting (see Table 1). The Coding Interactive Behavior (CIB) [38] observational system was used by a certified assessor to code the video-taped interaction. Children’s joint attention (i.e. the child’s gaze directed at the parent or object of joint attention) was used in this study. Children’s joint attention was coded on a 5-point rating scale, with 1 indicating low levels of joint attention and 5 indicating high levels of joint attention. Interrater reliability was good with an intraclass correlation coefficient of 0.76, based on double coded videos (21% of total sample).

#### Cognitive, motor, and language development at 24 months

Cognitive, motor, and language development were examined with the Dutch version of the Bayley-III (Bayley-III-NL) [39]. Bayley-III consists of five developmental scales: cognition, fine motor, gross motor, receptive language, and expressive language. Scaled scores were based on Dutch norms with means of 10 and SDs of 3 with good reliability and validity. Higher scores indicate better performance [39].

#### Behavioral problems at 24 months

Mothers completed the Child Behavior Checklist (CBCL/1.5–5) [40] to evaluate behavioral problems. The CBCL/1.5–5 comprises seven syndrome scales: emotionally reactive, anxious/depressed, somatic problems, withdrawn, sleep problems, attention problems, and aggressive behavior. The CBCL/1.5–5 consists of 100 items of problem behaviors for which mothers indicated to what extent children exhibit these behaviors on a 3-point rating scale (1 ‘not/never’, 2 ‘somewhat/sometimes’, 3 ‘very/often’). CBCL/1.5–5 standardized T-scores are truncated at a mean of 50, eliminating the lower half of the distribution and reporting low behavioral problems scores simply as a T-score of 50, resulting in standardized group means not below the mean T-score of 50. Nonetheless, standardized T-scores were used because they are based on Dutch age and sex norms with good reliability and validity [40].

#### Attention profiles at 6 years

A battery of eight neuropsychological tasks and two behavioral assessments was administered across two visits to assess multiple aspects of attentional functioning. Processing speed IQ (PSQ) was examined with two standardized subtests (Coding and Symbol Search) from the Wechsler Preschool and Primary Scale of Intelligence (WPPSI-III-NL). Scores were based on Dutch norms with means of 100 and SDs of 15 with good reliability and validity [41].

Four measures of processing speed and attention skills were assessed with the Cognitive Task Application (COTAPP), which is a computerized task with a total duration of 30–35 minutes: reaction time, variability in reaction time, inattention, and sustained attention. Reaction time is the mean reaction time across all tasks in which children were required to respond fast and accurately. Variability in reaction time is the (in)stability of these reaction times, assessed by the intra-individual coefficient of variance. Inattention is the number of extremely slow responses across all tasks, defined as responses slower than the child’s median reaction time + 3 SDs. Sustained attention is the difference in reaction times of identical tasks at the start and the end of the COTAPP. Scores were standardized z-scores derived from Dutch norms with moderate to good reliability (split-half: *r* = .59-.95; test-retest: *r* = .37-.85) and validity [42].

The Auditory attention subtest from the NEPSY-II is aimed at assessing auditory selective attention. Children were asked to listen to words on a 3-minute audio recording and point out a colored circle when hearing the corresponding color name, while ignoring other color names and irrelevant words (maximum number of correct responses = 20). The subtest has good test-retest reliability (*r* = .65) [43].

Two subtests from the Test of Everyday Attention in Children (TEA-Ch) were administered. The Sky search subtest is aimed at assessing visual selective attention. Children were asked to circle targets (pairs of identical space ships) while ignoring irrelevant targets (pairs of differing space ships) as fast as possible (maximum number of correct targets found = 20). For the Score! subtest, which is aimed at assessing auditory sustained attention, children were asked to count the number of tones on a 5.5-minute audio recording (maximum number of correct trials = 10). These subtests show moderate test-retest reliability (*r* = .57 and .72, respectively) [44].

Behavioral attention problems were assessed with the mother-reported attention problems syndrome scale (10 items) of the Child Behavior Checklist (CBCL/6-18) and teacher-reported inattention syndrome scale (14 items) of the Teacher Report Form (TRF/6-18). Standardized T-scores (attention problem syndrome scale) and percentiles (inattention syndrome scale) were derived from Dutch age and sex norms with good reliability and validity [45].

These 10 measures of attention were then used to derive three attention domains by principal component analysis: a) orienting attention and processing speed, b) alerting attention, and c) behavioral attention problems [16]. Principal component analysis scores were computed into standardized z-scores with lower scores indicating better performance. Table 3 presents the three attention domains or factors and their corresponding measures.

**Table 3:** Attention domains and corresponding measures.

Orienting attention & processing speed	Alerting attention	Behavioral attention problems
COTAPP Reaction time	TEA-Ch Sky search	TRF/6-18 Inattention
COTAPP Variability in reaction time	COTAPP Sustained attention	CBCL/6-18 Attention problems
COTAPP Inattention		
NEPSY Auditory attention		
TEA-Ch Score!		
WPPSI PSQ		

In our previous study, latent profile analysis revealed four distinct profiles of attentional functioning across these three attention domains. Based on children’s performance on these attention domains the analysis assigned them to one of the four previously described profiles: 1) Normal attention, 2) Overall poorer attention, 3) Poorer cognitive attention, and 4) Behavioral attention problems [16].

For the first aim of the study, early predictors for these four distinct attention profiles were evaluated. Given that some of the profiles were exhibited by small subgroups of children, we also dichotomized the four attention profiles into a group of normal attentional functioning (profile 1) and a group of subaverage attentional functioning (profiles 2, 3, or 4). For our second aim, early predictors were studied for normal attentional functioning versus subaverage attentional functioning groups.

### Statistical analyses

Participant characteristics (age at assessment, neonatal characteristics, medical characteristics at 6 years and sociodemographic characteristics) were examined across birth status groups (MLPT vs. FT) and attention profiles with one-way ANOVAs, chi-square tests, and Mann-Whitney U-tests. Twenty-six (15%) out of 170 participants seen at the 6-year follow-up had missing data on some of the measures at 18 and/or 24 months. The Expected-Maximization algorithm was performed to handle missing data for the remaining participants [46]. We examined whether attention, cognitive, motor, and language development, and behavioral problems at 18 and 24 months were associated with profile membership at 6 years, defined as one of the four distinct attention profiles [16]. Predictors at 18 and 24 months were first examined with three separate multinomial logistic regression models. Model 1 included measures of attention (UTATE and CIB), Model 2 included measures of cognitive, motor, and language development (Bayley-III-NL), and Model 3 comprised measures of behavioral problems (CBCL/1.5–5). Significant predictors from the three separate models were then entered in one final multinomial logistic regression model in relation to the four distinct attention profiles at 6 years. Considering that attention profiles were related to preterm birth and maternal education [16], the final model was also examined adjusted for birth status (MLPT vs. FT) and maternal education.

In addition, we examined whether these three sets of predictors were associated with profile membership at 6 years, dichotomized into normal attentional functioning (profile 1) versus subaverage attentional functioning (profiles 2, 3, and 4). Again three separate multivariable multinomial logistic regression models were conducted, from which significant predictors were entered into one final model. The final models for the four distinct attention profiles and dichotomized attention profiles were assessed with Cox & Snell *R*^*2*^ and model fit chi square-tests, and individual predictors at 18 and 24 months were examined using Wald tests and odds ratios.

## Results

### Participant characteristics

**Fig 1:**
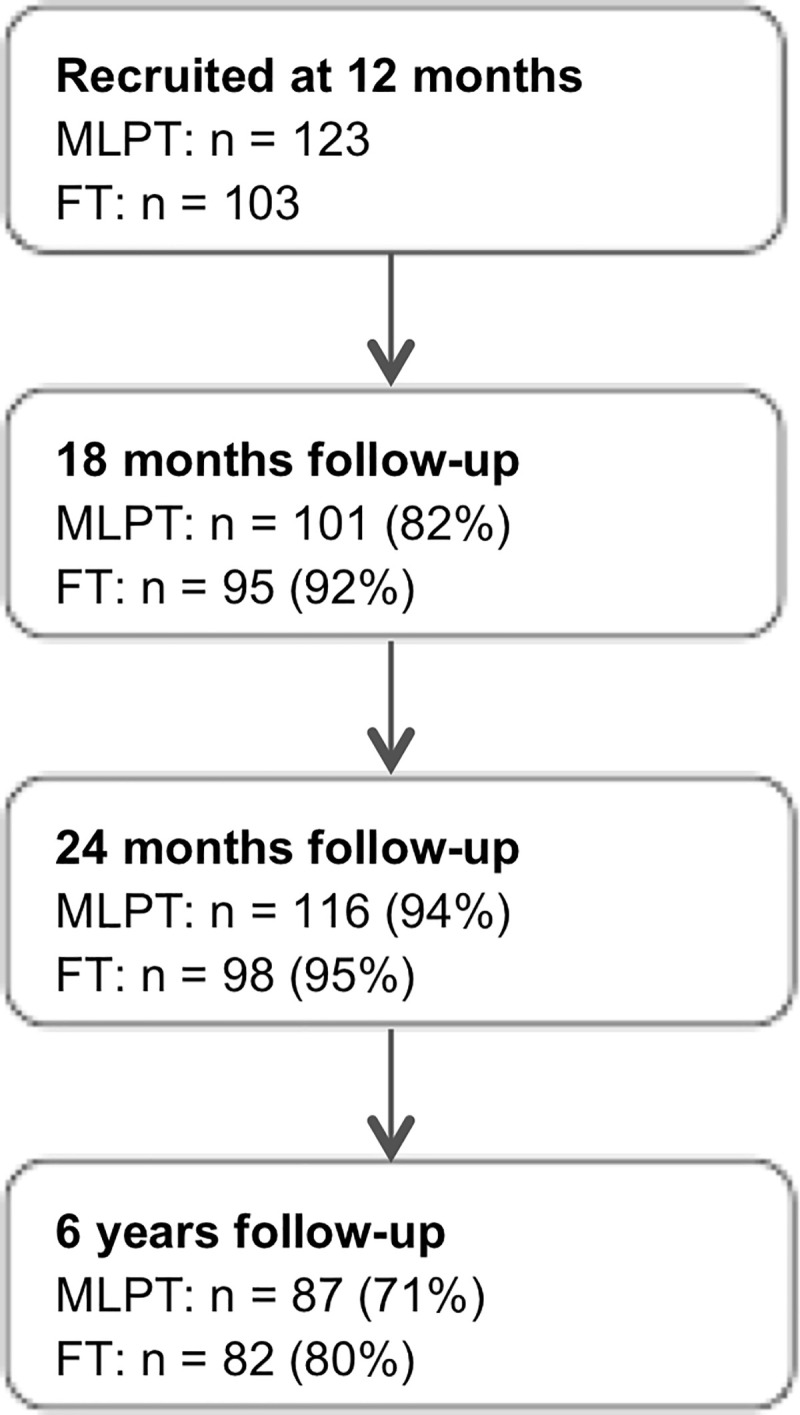
Study flowchart.

Participants’ inclusion and retention rates are shown in Fig 1. The present study included children who underwent comprehensive assessment of attentional functioning at 6 years of age, and for whom any data was available at 18 or 24 months. One participant did not have data on any of the measures at 18 and 24 months and was therefore excluded from the study. Children who dropped out at the 6 year follow-up did not differ from participants in terms of birth status, GA, birth weight, gender and maternal education. The final sample comprised 169 children, of which 82 were born full term and 87 moderate-to-late preterm. Table 4 shows participants’ neonatal and demographic characteristics per birth status group. Table 5 shows participants’ neonatal and demographic characteristics per profile, as well as for participants with subaverage attentional functioning (profiles 2, 3 and 4) combined. Children with a visual or hearing impairment (Tables 4 and 5) were included in the present study, because they were able to complete all tasks.

**Table 4:** Participant characteristics per birth status group (N = 169).

	FT	MLPT	
	(n = 82)	(n = 87)	*p*-value
	*M (SD)*	*M (SD)*	* *
*Age at assessment*			
Corrected age at 1^st^ assessment (months)	17.54 (0.50)	17.54 (0.50)	.92
Corrected age at 2^nd^ assessment (months)	23.68 (0.52)	23.56 (0.54)	.15
Corrected age at 3^rd^ assessment (years)	6.07 (0.06)	6.05 (0.05)	.08
*Neonatal characteristics*			
Gestational age (weeks)	39.54 (0.95)	34.67 (1.36)	< .001
Birth weight in grams	3605 (453)	2523 (492)	< .001
Birht weight centile category (*Mdn*)	10–16	80	< .001
Days in hospital	0.40 (1.06)	11.86 (10.14)	< .001
Need for oxygen^a^ (%)	0	23 (26%)	< .001
Phototherapy (%)	0	30 (35%)	< .001
Hypoglycemia (%)	0	4 (5%)	.048
Sex (% boys)	36 (44%)	50 (58%)	.08
*Medical characteristics at 6 years*			
Cerebral Palsy (%)	0	0	
Visual impairment partially corrected with aids (%)	0	1 (1%)	.33
Hearing impairment^b^ (%)	0	1 (1%)	.33
*Sociodemographic characteristics*			
Maternal education			< .001
Low^c^ (%)	2 (2%)	7 (8%)	
Medium^d^ (%)	8 (10%)	30 (35%)*	
High^e^ (%)	72 (88%)	50 (58%)*	

^a^Additional oxygen right after birth, nasal cannula and/or continuous positive airway pressure (CPAP).

^b^Single-sided deafness.

^c^Low = no education, elementary school, special education or lower general secondary education.

^d^Medium = secondary education or vocational education.

^e^High = college, university or higher.

**Table 5:** Participant characteristics per profile (N = 169).

Profile	1	2	3	4		2, 3, & 4	Comparison
	Normal	Overall poorer	Poorer cognitive	Behavioral	Comparison all	Subaverage	average vs.
	attention	attention	attention	attention problems	four profiles	attention	subaverage profiles
	n = 116	n = 13	n = 35	n = 5	*p*-value	n = 53	*p*-value
	*M (SD)*	*M (SD)*	*M (SD)*	*M (SD)*	* *	*M (SD)*	* *
*Latent profile components (attention domains)*							
Orienting attention & processing speed (z-score)	-0.25 (0.90)^f,g^	0.67 (0.68)^f^	0.54 (1.15)^g^	0.30 (0.74)	< .001	0.55 (1.01)	< .001
Alerting attention (z-score)	-0.50 (0.59)^f,g,h^	0.44 (0.84)^f,i^	1.46 (0.63)^g,i,k^	0.24 (0.27)^h,k^	< .001	1.09 (0.84)	< .001
Behavioral attention problems (z-score)	-0.34 (0.31)^f,h^	1.75 (0.57)^f,i,j^	-0.13 (0.45)^i,k^	4.25 (0.89)^h,j,k^	< .001	0.74 (1.49)	< .001
*Age at assessment*							
Corrected age at 1^st^ assessment (months)	17.54 (0.50)	17.45 (0.52)	17.60 (0.50)	17.40 (0.55)	.75	17.55 (0.50)	.87
Corrected age at 2^nd^ assessment (months)	23.65 (0.51)	23.54 (0.52)	23.57 (0.56)	23.60 (0.89)	.82	23.57 (0.57)	.35
Corrected age at 3^rd^ assessment (years)	6.07 (0.06)	6.05 (0.05)	6.05 (0.06)	6.06 (0.06)	.47	6.06 (0.06)	.33
*Neonatal characteristics*							
Birth status (% MLPT)	51 (44%)^f,g^	10 (77%)^f^	22 (63%)^g^	4 (80%)	.02	36 (68%)	.004
Gestational age (weeks)	37.48 (2.59)	35.08 (3.20)	36.46 (2.49)	35.60 (2.88)	.004	36.04 (2.72)	.001
Birth weight (grams)	3165 (682)^g^	2776 (1138)	2842 (573)^g^	2518 (559)	.01	2795 (739)	.002
Birth weight centile category (*Mdn*)	50	80	20–50	50	.37	50	.58
Days in hospital	5.01 (8.24)	14.00 (12.61)	7.03 (9.27)	11.00 (13.64)	.005	9.11 (10.79)	.007
Need for oxygen^a^ (%)	12 (10%)^f^	5 (39%)^f^	5 (14%)	1 (20%)	.045	11 (21%)	.07
Phototherapy (%)	15 (14%)	3 (23%)	10 (29%)	1 (20%)	.23	14 (26%)	.046
Hypoglycemia (%)	3 (3%)	0	1 (3%)	0	.92	1 (2%)	.78
Sex (% boys)	53 (46%)	11 (85%)	19 (54%)	3 (60%)	.06	33 (62%)	.046
*Medical characteristics at 6 years*							
Cerebral Palsy	0	0	0	0		0	
Visual impairment partially corrected with aids (%)	0^f^	1 (8%)^f,i,j^	0^i^	0^j^	.007	1 (2%)	.14
Hearing impairment^b^ (%)	1 (1%)	0	0	0	.93	0	.50
*Sociodemographic characteristics*							
Maternal education					.003		.003
Low^c^ (%)	4 (4%)^g^	0 (0%)	5 (14%)^g^	0 (0%)		5 (9%)	
Medium^d^ (%)	19 (16%)^f^	7 (54%)^f^	11 (32%)	1 (20%)		19 (36%)*	
High^e^ (%)	93 (80%)^f,g^	6 (46%)^f^	19 (54%)^g^	4 (80%)		29 (55%)*	

^a^Additional oxygen right after birth, nasal cannula and/or continuous positive airway pressure (CPAP).

^b^Single-sided deafness.

^c^Low = no education, elementary school, special education or lower general secondary education.

^d^Medium = secondary education or vocational education.

^e^High = college, university or higher.

Pairwise comparison p < .05: Profile f1 versus 2; g1 versus 3; h1 versus 4; i2 versus 3; j2 versus 4; k3 versus 4.

Table 5 shows that children with the Normal attention profile (1; n = 117) had average attentional functioning on all three attention domains. Children with the Overall poorer attention profile (2; n = 13) performed poorer across all three attention domains. Children classified in the Poorer cognitive attention profile (3; n = 35) showed substantially poorer performance in alerting attention, and poorer orienting attention and processing speed, but not in the behavioral attention domain. Finally, children in the Behavioral attention problems profile (4; n = 5) showed very poor performance on the behavioral attention domain (i.e. parent- and/or teacher-rated behavioral attention problems) and somewhat poorer alerting attention, but average orienting attention and processing speed performance.

### Correlations between predictors

Significant correlations between predictors from different measurement tools at 18 and 24 months were generally very weak (*r* = 0.15–0.28). Within the UTATE significant correlations were moderate to very strong (*r* = 0.34–0.85). Within the Bayley-III-NL significant correlations were weak to moderate (*r* = 0.24–0.55). Within the CBCL/1.5–5 significant correlations were very weak to moderate (*r* = 0.16–0.54).

### Distinct attention profiles

Table 6 presents descriptive statistics per profile for measures of attention at 18 months, and cognitive, motor, and language development, and behavioral problems at 24 months.

**Table 6:** Means and SDs of predictors at 18 and 24 months per attention profile at 6 years (N = 169).

Profile	1	2	3	4	2, 3, & 4
	Normal	Overall poorer	Poorer cognitive	Behavioral	Subaverage
	attention	attention	attention	attention problems	attention
	n = 116	n = 13	n = 35	n = 5	n = 53
	*M (SD)*	*M (SD)*	*M (SD)*	*M (SD)*	*M (SD)*
Attention					
Orienting attention (UTATE)	0.06 (0.35)	-0.07 (0.24)	-0.20 (0.43)	0.03 (0.62)	-0.15 (0.41)
Alerting attention (UTATE)	0.08 (0.53)	0.06 (0.42)	-0.32 (0.72)	0.37 (0.51)	-0.16 (0.67)
Executive attention (UTATE)	0.00 (0.63)	0.07 (0.61)	-0.07 (0.67)	0.53 (0.57)	0.02 (0.66)
Joint attention (CIB)	3.51 (0.94)	3.18 (0.80)	3.11 (0.87)	3.00 (0.71)	3.12 (0.82)
General development (Bayley-III)					
Cognition	10.66 (2.13)	10.38 (2.36)	9.43 (2.37)	9.40 (3.05)	9.66 (2.42)
Fine Motor	11.91 (1.83)	11.92 (2.67)	10.80 (2.61)	11.20 (3.35)	11.11 (2.68)
Gross Motor	9.41 (2.72)	9.46 (3.53)	9.14 (2.94)	10.00 (3.39)	9.30 (3.08)
Receptive Language	11.96 (2.73)	10.00 (2.41)	10.60 (2.66)	11.80 (2.59)	10.57 (2.59)
Expressive Language	11.35 (2.34)	10.92 (2.14)	10.77 (3.13)	11.60 (1.52)	10.89 (2.77)
Behavioral problems (CBCL/1.5–5)					
Emotionally reactive	52.06 (4.13)	51.71 (2.73)	52.46 (4.21)	54.03 (4.56)	52.42 (3.91)
Anxious/depressed	50.54 (1.35)	50.20 (0.39)	50.88 (2.24)	50.04 (0.09)	50.63 (1.86)
Somatic complaints	52.50 (4.53)	52.75 (3.27)	54.50 (5.69)	50.68 (1.52)	53.71 (5.04)
Withdrawn	52.09 (3.17)	51.52 (2.13)	52.18 (3.57)	51.57 (2.53)	51.96 (3.16)
Sleep problems	51.91 (3.75)	52.67 (3.57)	53.23 (4.72)	50.02 (0.69)	52.79 (4.29)
Attention problems	52.87 (4.68)	54.03 (5.85)	52.50 (4.12)	53.72 (4.95)	52.99 (4.62)
Aggressive behavior	52.46 (4.10)	54.85 (5.08)	52.39 (3.80)	51.95 (2.53)	52.95 (4.13)

Associations of attention at 18 months (Model 1), cognitive, motor, and language development at 24 months (Model 2), and behavioral problems at 24 months (Model 3) with distinct attention profiles at 6 years are shown in odds ratios in Table 7. In Model 1, orienting and alerting attention at 18 months were significant predictors of distinct attention profiles at 6 years (Cox & Snell *R^2^* = .18, *χ^2^*(12) = 34.10, *p* = .001). In Model 2, receptive language at 24 months was a significant predictor of distinct attention profiles at 6 years (Cox & Snell *R^2^* = .13, *χ^2^*(15) =, *p* = .09). In Model 3, emotionally reactive behavior and sleep problems at 24 months were significant predictors of distinct attention profiles at 6 years (Cox & Snell *R^2^* = .18, *χ^2^*(21) = 34.37, *p* = .03).

**Table 7:** Predictors of distinct attention profiles at 6 years (N = 169).

* *	Overall poorer	Poorer cognitive	Behavioral attention problems	Poorer cognitive	Behavioral attention problems	Behavioral attention problems
	vs. Normal attention	vs. Normal attention	vs. Normal attention	vs. Overall poorer	vs. Overall poorer	vs. Poorer cognitive
**Predictors**	OR [95% CI]	OR [95% CI]	OR [95% CI]	OR [95% CI]	OR [95% CI]	OR [95% CI]
Attention (Model 1)						
Orienting attention	0.02 [0.00, 0.77]*	0.39 [0.04, 3.76]	0.001 [0.00, 0.60]*	19.70 [0.39, 998.88]	0.07 [0.00, 51.15]	0.01 [0.00, 1.75]^#^
Alerting attention	10.15 [0.67, 154.08]^#^	0.40 [0.08, 1.99]	174.30 [0.96, 31603.35]^#^	0.04 [0.00, 0.74]*	17.17 [0.06, 4819.27]	433.84 [2.09, 90046.06]*
Executive attention	0.88 [0.25, 3.12]	1.85 [0.82, 4.17]	2.14 [0.30, 15.44]	2.09 [0.52, 8.48]	2.43 [0.26, 22.89]	1.16 [0.15, 9.13]
Joint attention	0.62 [0.32, 1.20]	0.67 [0.42, 1.07]^#^	0.39 [0.13, 1.21]	1.08 [0.52, 2.25]	0.63 [0.18, 2.17]	0.59 [0.18, 1.88]
General development (Model 2)						
Cognition	1.02 [0.74, 1.40]	0.83 [0.66, 1.04]	0.68 [0.39, 1.19]	0.82 [0.57, 1.17]	0.67 [0.36, 1.25]	0.82 [0.46, 1.46]
Fine Motor	1.07 [0.76, 1.50]	0.85 [0.69, 1.05]	0.89 [0.55, 1.42]	0.80 [0.55, 1.15]	0.83 [0.47, 1.47]	1.05 [0.64, 1.71]
Gross Motor	1.05 [0.83, 1.32]	1.08 [0.93, 1.26]	1.18 [0.85, 1.62]	1.03 [0.80, 1.33]	1.12 [0.77, 1.64]	1.09 [0.78, 1.52]
Receptive Language	0.71 [0.54, 0.93]*	0.85 [0.70, 1.03]^#^	0.99 [0.62, 1.58]	1.20 [0.89, 1.63]	1.40 [0.83, 2.37]	1.17 [0.72, 1.89]
Expressive Language	1.10 [0.80, 1.51]	1.11 [0.89, 1.39]	1.19 [0.68, 2.07]	1.01 [0.71, 1.44]	1.08 [0.58, 2.02]	1.07 [0.60, 1.90]
Behavioral problems (Model 3)						
Emotionally reactive	0.92 [0.74, 1.14]	0.99 [0.87, 1.13]	2.08 [1.18, 3.66]*	1.08 [0.84, 1.37]	2.25 [1.24, 4.10]**	2.10 [1.18, 3.73]*
Anxious/depressed	0.54 [0.15, 1.96]	1.12 [0.85, 1.46]	0.02 [0.00, 1.89]^#^	2.08 [0.56, 7.68]	0.43 [0.00, 4.16]	0.02 [0.00, 1.70]^#^
Somatic complaints	1.04 [0.89, 1.20]	1.08 [0.99, 1.17]	0.62 [0.23, 1.65]	1.04 [0.89, 1.22]	0.60 [0.22, 1.61]	0.58 [0.26, 1.54]
Withdrawn	0.87 [0.66, 1.15]	0.97 [0.84, 1.12]	0.64 [0.30, 1.39]	1.11 [0.83, 1.51]	0.74 [0.33, 1.66]	0.66 [0.30, 1.45]
Sleep problems	1.03 [0.89, 1.19]	1.07 [0.97, 1.17]	0.14 [0.02, 0.88]*	1.03 [0.89, 1.21]	0.13 [0.02, 0.86]*	0.13 [0.02, 0.83]*
Attention problems	0.96 [0.84 1.10]	0.96 [0.86, 1.08]	1.43 [0.98, 2.10]	1.01 [0.85, 1.19]	1.49 [1.00, 2.24]	1.49 [1.00, 2.20]
Aggressive behavior	1.23 [1.03, 1.47]^#^	0.97 [0.84, 1.11]	0.69 [0.38, 1.26]	0.79 [0.64, 0.97]^#^	0.57 [0.31, 1.05]^#^	0.72 [0.39, 1.32]
Final model (unadjusted)						
Orienting attention	0.01 [0.00, 0.62]*	0.23 [0.03, 2.20]	0.004 [0.00, 0.62]*	16.63 [0.29, 957.65]	0.26 [0.00, 99.39]	0.02 [0.00, 3.34]
Alerting attention	11.75 [1.15, 120.35]*	0.80 [0.21, 3.02]	94.54 [1.67, 5340.63]*	0.07 [0.01, 0.82]*	8.04 [0.10, 659.17]	118.24 [1.85, 7566.80]*
Receptive Language	0.74 [0.59, 0.94]*	0.86 [0.75, 1.00]^#^	0.87 [0.58, 1.32]	1.16 [0.90, 1.49]	1.17 [0.74, 1.85]	1.01 [0.66, 1.55]
Emotionally reactive	0.92 [0.77, 1.11]	0.97 [0.88, 1.08]	1.14 [0.90, 1.43]	1.05 [0.87, 1.27]	1.23 [0.92, 1.64]	1.17 [0.91, 1.50]
Sleep problems	1.04 [0.90, 1.21]	1.06 [0.96, 1.16]	0.21 [0.01, 3.90]	1.01 [0.87, 1.18]	0.20 [0.01, 3.75]	0.19 [0.01, 3.69]
Final model (adjusted)^a^						
Orienting attention	0.01 [0.00, 0.61]*	0.20 [0.02, 1.95]	0.01 [0.00, 1.67]^#^	16.15 [0.26, 997.27]	0.70 [0.00, 314.91]	0.04 [0.00, 10.80]
Alerting attention	16.85 [1.49, 190.47]*	1.10 [0.28, 4.37]	59.03 [1.09, 3210.95]*	0.07 [0.01, 0.84]*	3.50 [0.04, 285.69]	53.54 [0.86, 3334.69]^#^
Receptive Language	0.76 [0.60, 0.97]*	0.87 [0.74, 1.01]^#^	0.89 [0.57, 1.40]	1.14 [0.88, 1.48]	1.17 [0.71, 1.93]	1.03 [0.65, 1.64]
Emotionally reactive	0.89 [0.74, 1.08]	0.96 [0.86, 1.06]	1.11 [0.86, 1.42]	1.07 [0.88, 1.31]	1.24 [0.91, 1.68]	1.16 [0.89, 1.51]
Sleep problems	1.05 [0.90, 1.43]	1.05 [0.96, 1.16]	0.24 [0.01, 4.12]	1.01 [0.87, 1.17]	0.23 [0.01, 3.95]	0.22 [0.01, 3.92]

^a^Adjusting the model for gestational age instead of birth status yielded similar results. Results not shown.

**p < .01

*p < .05

^#^*p* < .10.

Significant predictors from Models 1, 2 and 3 (i.e. orienting attention, alerting attention, receptive language, emotionally reactive behavior, and sleep problems) were entered as predictors in the final model (Cox & Snell *R^2^* = .22, *χ^2^*(15) = 41.85, *p* < .001). As shown in Table 7 (see Final model unadjusted), orienting and alerting attention at 18 months, and receptive language at 24 months were significant predictors of attention profile membership at 6 years. Compared to the Normal attention profile (1), the Overall poorer attention profile (2) was predicted by lower orienting attention and lower receptive language, and by somewhat higher alerting attention. In addition, the Overall poorer profile (2) was distinguished from the Poorer cognitive attention profile (3) by higher alerting attention.

As presented in Table 7, the Poorer cognitive attention profile (3) was not distinguished significantly from the Normal attention profile (1) by any of the predictors. The Poorer cognitive attention profile (3) was, however, predicted by lower alerting attention compared with the Overall poorer attention (2) and Behavioral attention problems (4) profiles.

Finally, compared with the Normal attention profile (1), the Behavioral attention problems profile (4) was predicted by lower orienting attention and higher alerting attention. Additionally, the Behavioral attention problems profile (4) was distinguished from the Poorer cognitive attention (3) by higher alerting attention.

Because birth status (MLPT vs. FT) and maternal education differed across attention profiles, the final model was also examined adjusting for birth status (MLPT vs. FT) and maternal education (Table 7; see Final model adjusted). To avoid collinearity, the model was not adjusted for gestational age and birth weight in addition to birth status. After adjusting, results regarding orienting attention were largely unaltered. The only change was that orienting attention as a predictor of the Behavioral attention profile (4) compared to the Normal attention profile (1) attenuated to *p* = .08. For alerting attention results were unchanged, except for alerting attention as a predictor comparing the Behavioral attention profile (4) to the Poorer cognitive attention profile (3), which decreased to *p* = .059. The findings for receptive language remained the same.

### Normal versus subaverage attentional functioning groups

Table 8 presents group means on attention at 18 months, and cognitive, motor, and language development, and behavioral problems at 24 months. In Table 8, profile 1. Normal attention represents the normal attentional functioning group, and profiles 2, 3 & 4. Subaverage attention represent the subaverage attentional functioning groups.

**Table 8:** Predictors of normal vs. subaverage attention profiles at 6 years (N = 169).

* *	Normal attention profile
	vs. Subaverage attention profiles
**Predictors**	OR [95% CI]
Attention (Model 1)	
Orienting attention	0.14 [0.02, 1.07]^#^
Alerting attention	1.20 [0.30, 4.84]
Executive attention	1.57 [0.78, 3.14]
Joint attention	0.63 [0.43, 0.94]*
General development (Model 2)	
Cognition	0.86 [0.71, 1.04]
Fine Motor	0.89 [0.74, 1.08]
Gross Motor	1.08 [0.95, 1.24]
Receptive Language	0.82 [0.70, 0.96]*
Expressive Language	1.12 [0.93, 1.35]
Behavioral problems (Model 3)	
Emotionally reactive	1.01 [0.90, 1.12]
Anxious/depressed	1.00 [0.78, 1.29]
Somatic complaints	1.05 [0.97, 1.13]
Withdrawn	0.95 [0.83, 1.08]
Sleep problems	1.04 [0.96, 1.14]
Attention problems	0.98 [0.90, 1.07]
Aggressive behavior	1.03 [0.93, 1.15]
Final model (unadjusted)	
Orienting attention	0.28 [0.11, 0.73]**
Joint attention	0.71 [0.48, 1.05]^#^
Receptive Language	0.87 [0.76, 0.99]*
Final model (adjusted)^a^	
Orienting attention	0.36 [0.13, 0.99]*
Joint attention	0.79 [0.52, 1.19]
Receptive Language	0.87 [0.76, 1.00]*

^a^Adjusting the model for gestational age instead of birth status yielded similar results. Results not shown.

**p < .01

*p < .05

^#^*p* < .10.

Associations of attention at 18 months (Model 1), cognitive, motor, and language development at 24 months (Model 2), and behavioral problems at 24 months (Model 3) with normal versus subaverage attentional functioning at 6 years are shown in odds ratios in Table 8. In Model 1, orienting attention and joint attention were significant predictors (Cox & Snell *R^2^* = .11, *χ^2^*(5) = 18.98, *p* = .002). Orienting attention showed a significant likelihood ratio test (*χ^2^*(3) = 8.38, *p* = .048) and was therefore included in the final model, even though the odds ratio for orienting attention shown in Table 8 had a *p-*value of .058. In Model 2, receptive language was associated with a normal versus subaverage attentional functioning (Cox & Snell *R^2^* = .09, *χ^2^*(5) = 15.69, *p* = .008). In Model 3, no significant associations were found between behavioral problems and normal versus subaverage attentional functioning (Cox & Snell *R^2^* = .02, *χ^2^*(7) = 4.12, *p* = .77).

Significant predictors from Models 1, 2, and 3 (i.e. orienting attention, joint attention, and receptive language) were then entered as predictors in the final model (Cox & Snell *R^2^* = .12, *χ^2^*(3) = 20.99, *p* < .001). As shown in Table 8 (Final model unadjusted), lower orienting attention at 18 months and lower receptive language at 24 months predicted subaverage attentional functioning at 6 years. After adjusting for birth status and maternal education, results remained unchanged (Table 8; Final model adjusted).

## Discussion

The main aim of this study was to explore and identify potentially important predictors at toddler age of distinct attention profiles or subaverage attentional functioning at school-age. In the present study, attention abilities, cognitive, motor, and language development, and behavioral problems at toddler age were evaluated in a sample of children born MLPT and FT. Overall, we found that orienting and alerting attention at 18 months, and receptive language at 24 months were related to distinct attention profiles at 6 years. Findings of associations between these toddler abilities and attention profiles at 6 years were fairly robust, with the majority of associations remaining unchanged after adjusting for birth status and maternal education. Thus, while MLPT children are at increased risk of poorer attentional functioning compared with FT children [16], birth status in itself is insufficient as a predictor given that attention and language skills at toddler age are also related to school-age attentional functioning even after adjusting for birth status.

Relationships between toddler abilities and attentional functioning were examined using two approaches. First, we examined relationships with the four distinct attention profiles, and in the second approach we regarded the relationships of toddler abilities with normal attentional functioning (profile 1) versus subaverage attentional functioning (profiles 2, 3, and 4). While it should be noted that some subgroups had a small sample size in the first approach, the associations found between toddler abilities and attentional functioning at 6 years were similar in both approaches. Orienting attention, alerting attention and receptive language at toddler age were associated with distinct attention profiles, and orienting attention and receptive language were related to normal versus subaverage attentional functioning. Toddlers performing poorer on orienting attention as well as receptive language, were at increased risk for subaverage attentional functioning at 6 years. More differentiated findings appeared when all four profiles were examined, even though these findings should be interpreted cautiously due to small subgroups. Although children with the Normal attention profile showed highest performance on orienting attention and receptive language, children with a subaverage attention profile did not all perform significantly poorer on these toddler abilities. Lower orienting attention specifically differentiated children with the Overall poorer attention and the Behavioral attention problems profiles from children with the Normal attention profile. For receptive language, poorer performance specifically distinguished children with the Overall poorer attention profile from peers with the Normal attention profile.

The relationship between early orienting attention and later distinct attention profiles was not unexpected, given that both constructs are aspects of attentional functioning. The association we found between receptive language, i.e. language comprehension, at toddler age and attentional functioning at school-age may, however, reflect more indirect relationships. This finding may indicate that attention and language share mutual underlying factors, such as child regulation, general cognitive functioning, or parent-infant interaction quality [47, 48]. Alternatively, attention abilities may be a precursor for language functioning [27, 49], given that attention is an elementary skill. Better attention abilities may help children engage in social interactions and maintain focus during verbalizations, enabling more optimal language development [50]. Indeed, one study demonstrated that children’s alerting attention abilities combined with parent’s word naming at 9 months predicted later language comprehension at 12 and 15 months [51]. As our results show that both early attention and receptive language contribute to later attentional functioning, it is likely that bidirectional relationships are involved.

Our findings for early alerting attention skills were more complex. Alerting attention was not predictive of normal versus subaverage attentional functioning at 6 years, because performance differed considerably across the distinct attention profiles. Alerting attention at toddler age differentiated the Poorer cognitive attention profile from the Normal attention and Overall poorer attention profiles, as well as the Behavioral attention problems profile from the Normal attention profile. Children who at 6 years had a Poorer cognitive attention profile, which was characterized by substantially poorer alerting attention [16], already showed shorter looking durations for visually presented stimuli in our eye tracking task (the UTATE) at 18 months. These shorter looking durations indicate more difficulty with maintaining focus (i.e. alerting attention). Thus, these children with a Poorer cognitive attention profile could already be differentiated by poorer alerting attention as toddlers. Conversely, children with the Behavioral attention problems profile were differentiated by higher alerting attention scores at 18 months, while at the age of 6 years these children showed slightly poorer alerting attention [16]. In other words, these children who at 6 years had poorer alerting attention, reflected by less accurate responses and slowing of reaction times after focusing for a considerable period, exhibited longer looking durations for visual information at toddler age. Typically, better alerting attention is regarded as the ability to maintain focus for a longer duration. However, the toddler and school-age measures of alerting attention differed. Unlike the alerting attention tasks for older children, our eye tracking task at 18 months evaluated children’s looking behavior without requiring further (verbal or motor) response, to avoid confounding attention with other abilities at this young age. A disadvantage to this approach is that it is not directly apparent whether a child has accurately processed the visual information, or if notably longer looking durations may rather indicate inattention or absent-mindedness. Further research in typically developing children is needed to investigate whether toddlers’ longer looking durations indeed reflect optimal alerting attention, or whether this operationalization follows a U-shaped curve instead, with optimal alerting attention manifested by looking durations centered around the mean. In addition, other types of measures of alerting attention at toddler age may be designed and tested for validity.

Several implications can be derived from the present exploratory study. The most important finding is that difficulties in early orienting and alerting attention, and receptive language may have distinctive implications for (both MLPT and FT) children’s attentional functioning at school-age, and may result in different attention difficulties. The two approaches we used (examining relationships with four distinct attention profiles as well as with normal versus subaverage attentional functioning) showed similar results, yet also provide cautious support for the value and importance of differentiating distinct attention profiles. Our results indicate that children who were classified in distinct attention profiles at 6 years, exhibit differentiated functioning in orienting and alerting attention, and language comprehension already at toddler age. As our previous study showed that MLPT birth increased the risk of poorer attentional functioning [16], monitoring programs for children at risk of attention difficulties, such as children born preterm, should ideally include specific early attention measures as well as measures of language comprehension at toddler age. In other words, children at risk of attention difficulties, such as MLPT children, need to be further identified with early assessments of attention and language skills. For such programs, assessment of toddlers attentional functioning on multiple attention abilities is warranted, because difficulties in specific attention abilities may have a different impact on functioning in daily life. Given that orienting and alerting attention at toddler age are markers for later attention difficulties, both monitoring and interventions should be implemented already before school-age to improve long-term attentional functioning. As child abilities are differentiated already at toddler age, interventions should be tailored to individual needs, making use of an integrated framework of child development, parent involvement, and therapist support, as recommended by other research [52, 53].

A limitation of our study is that some of the profiles had small subgroup sizes or high variance in toddler abilities, requiring replication with larger sample sizes. Nevertheless, when we examined toddler abilities in relation to normal attentional functioning versus subaverage attentional functioning, comprising larger subgroup sizes, similar associations of toddler abilities, specifically orienting attention and receptive language, with attentional functioning were still demonstrated. Moreover, because this was an exploratory study aimed at identifying potentially important predictors at toddler age of later attentional functioning, we examined a relatively broad range of toddler skills and measurement tools. Further research is needed to investigate the underlying mechanisms of the associations found in this study. In particular, it is important to examine and eventually target factors that may impact early attention and language skills in children at risk for poorer attentional functioning at school-age, such as MLPT children. Strengths of the present study include the longitudinal design with repeated, multidimensional assessment of attention constructs over time, both general and specific measures of toddler abilities, and use of multiple informants. As such, this study extends previous research aiming to differentiate children’s variation in attentional functioning, in particular for children at risk of attention difficulties. Already at toddler age, differentiated functioning in early attention abilities and in language comprehension predicted distinct attention profiles at school-age. To conclude, our study shows that distinct attention profiles at school-age, along with considering normal versus subaverage attentional functioning, can be predicted from assessments of specific attention abilities as well as language abilities. Therefore, our study highlights the need for monitoring of at-risk children’s attention and language abilities in children at risk from an early age until at least school-age.
